# Targeting Telomere Biology in Acute Lymphoblastic Leukemia

**DOI:** 10.3390/ijms22136653

**Published:** 2021-06-22

**Authors:** Axel Karow, Monika Haubitz, Elisabeth Oppliger Leibundgut, Ingrid Helsen, Nicole Preising, Daniela Steiner, Tobias M. Dantonello, Roland A. Ammann, Jochen Roessler, Mutlu Kartal-Kaess, Alexander Röth, Gabriela M. Baerlocher

**Affiliations:** 1 Department of Pediatrics, Division of Pediatric Hematology/Oncology, Inselspital, Bern University Hospital, University of Bern, 3012 Bern, Switzerland; axel.karow@uk-erlangen.de (A.K.); tobias.dantonello@insel.ch (T.M.D.); Roland.Ammann@insel.ch (R.A.A.); jochen.roessler@insel.ch (J.R.); mutlu.kartal-kaess@insel.ch (M.K.-K.); 2 Pediatric Hematology/Oncology, Department of BioMedical Research (DBMR), University of Bern, 3012 Bern, Switzerland; 3 Laboratory for Hematopoiesis and Molecular Genetics, Experimental Hematology, Department of BioMedical Research (DBMR), University of Bern, 3008 Bern, Switzerland; monika.haubitz@dbmr.unibe.ch (M.H.); Elisabeth.oppliger@insel.ch (E.O.L.); ingrid.helsen@dbmr.unibe.ch (I.H.); daniela.steiner@vetsuisse.unibe.ch (D.S.); 4 Department of Hematology and Central Hematology Laboratory, Inselspital, Bern University Hospital, University of Bern, 3012 Bern, Switzerland; 5 Department of Hematology and Stem Cell Transplantation, West German Cancer Center, University Hospital Essen, University of Duisburg-Essen, 47147 Essen, Germany; nicole.preising@uk-essen.de (N.P.); alexander.roeth@uk-essen.de (A.R.); 6 Kinderaerzte KurWerk, 3400 Burgdorf, Switzerland

**Keywords:** acute lymphoblastic leukemia (ALL), clonal expansion, replicative history, telomere length, telomerase activity, prognostic markers, telomerase inhibitor, imetelstat

## Abstract

Increased cell proliferation is a hallmark of acute lymphoblastic leukemia (ALL), and genetic alterations driving clonal proliferation have been identified as prognostic factors. To evaluate replicative history and its potential prognostic value, we determined telomere length (TL) in lymphoblasts, B-, and T-lymphocytes, and measured telomerase activity (TA) in leukocytes of patients with ALL. In addition, we evaluated the potential to suppress the in vitro growth of B-ALL cells by the telomerase inhibitor imetelstat. We found a significantly lower TL in lymphoblasts (4.3 kb in pediatric and 2.3 kb in adult patients with ALL) compared to B- and T-lymphocytes (8.0 kb and 8.2 kb in pediatric, and 6.4 kb and 5.5 kb in adult patients with ALL). TA in leukocytes was 3.2 TA/C for pediatric and 0.7 TA/C for adult patients. Notably, patients with high-risk pediatric ALL had a significantly higher TA of 6.6 TA/C compared to non-high-risk patients with 2.2 TA/C. The inhibition of telomerase with imetelstat ex vivo led to significant dose-dependent apoptosis of B-ALL cells. These results suggest that TL reflects clonal expansion and indicate that elevated TA correlates with high-risk pediatric ALL. In addition, telomerase inhibition induces apoptosis of B-ALL cells cultured in vitro. TL and TA might complement established markers for the identification of patients with high-risk ALL. Moreover, TA seems to be an effective therapeutic target; hence, telomerase inhibitors, such as imetelstat, may augment standard ALL treatment.

## 1. Introduction

Recently, a number of genomic studies have facilitated the further subclassification of pediatric and adult acute lymphoblastic leukemia (ALL) and provided deeper insight into the interplay of genetic alterations and their possible role in disease pathogenesis [1,2]. Some of these alterations bear significant implications for the diagnosis, risk stratification, and therapeutic approach of childhood and adult ALL [3,4]. However, early identification of patients with high-risk disease allowing timely treatment adaption remains challenging. Complementing prognostic markers and additional therapeutic agents are urgently needed.

Uncontrolled cellular proliferation is a key feature of ALL, and the assessment of altered regulatory cellular mechanisms driving clonal expansion might add to the understanding of different disease courses and the identification of potential therapeutic targets. Cellular proliferation in human cells is closely associated with the regulation of telomere length (TL) maintenance and telomerase activity (TA). Due to the lack of TA in most human somatic cells, telomere repeats are lost with each cell division, resulting in telomere attrition with cellular replication and age. In contrast, most human cancer cells reactivate telomerase, thereby compensating for the loss of telomere repeats with cellular replication, enabling immortality [5,6]. Short telomeres and detectable levels of TA are described in adult and pediatric solid tumors and hematologic neoplasia [7,8,9,10,11,12]. Telomerase has become a target of novel therapies, and recent preclinical and clinical studies have demonstrated the efficacy of the competitive telomerase inhibitor imetelstat in hematologic malignancies, in particular, myeloproliferative neoplasms, myelodysplastic syndromes, and acute myeloid leukemia [13,14,15,16].

The main aim of this pilot study was to test whether the determination of TL and TA at the time of diagnosis could facilitate the early identification of pediatric patients with ALL and high-risk features, allowing timely treatment adaption. In addition, our goal was to assess the ex vivo effects of imetelstat on primary B-ALL cells, suggesting a potential augmentative role in future ALL treatment.

## 2. Patients and Methods

### 2.1. Study Design

Screening of TL and TA was conducted in two separate patient cohorts, one comprising pediatric and the other, adult patients with ALL. In the latter cohort, additional assays testing the ex vivo effects of imetelstat on primary B-ALL cells were performed. The study was carried out in accordance with the Declaration of Helsinki after approval by the Swiss cantonal Ethics Committee, Bern (Ref.-Nr. KEK-BE: 2017-00914/2019-01043). Written informed consent was obtained from the patients’ legal guardians and the patients, if applicable, after they were provided age-appropriate oral and written information.

From the pediatric patients, between 2.5 mL and 5 mL of EDTA-anticoagulated peripheral blood (PB) was collected in parallel with routine diagnostics for the measurements of TL in leukocyte subsets at the time of diagnosis, and of TA in leukocytes at the time of diagnosis and during induction therapy.

Clinical and laboratory data at diagnosis and during follow up were extracted from in-house and reference records.

For measurements of TL and TA and testing of the ex vivo effects of imetelstat in primary cells of adult patients with B-ALL, samples with 10 mL of EDTA-anticoagulated PB were collected at the Department of Hematology, University Hospital Essen, Germany, after obtaining informed consent and according to institutional guidelines.

### 2.2. Patients

For the pediatric cohort, children and adolescents aged 0–17 years with newly diagnosed B- or T-ALL before treatment initiation were eligible. All pediatric patients were treated according to the international collaborative treatment protocol for children and adolescents with ALL of the AIEOP-BFM ALL 2009 Registry. Accordingly, standard induction therapy comprised the continuous application of oral steroids from day 1 with tapering between days 29 and 37; infusion of vincristine and daunorubicin on days 8, 15, 22, and 29; and PEG-asparaginase on days 12 and 26, complemented by intrathecal methotrexate on days 1, 12, and 33.

For the adult cohort, patients ≥ 18 years with a diagnosis of B-ALL before the start of therapy were included. Classification into risk groups was based on hematologic parameters, immunophenotype, (cyto)genetics, and treatment response [4,17].

### 2.3. Measurement of Telomere Length

Blood samples were transferred immediately to the laboratory, and an erythrocyte lysing step, using ammonium chloride (STEMCELL Technologies Germany, GmbH, Cologne, Germany) added by a washing step in PBS (pH 7.3, no calcium, no magnesium; Institute of Hospital Pharmacy, Bern University Hospital, Bern, Switzerland), was performed to obtain leukocytes for subsequent analyses. Leukocytes were then counted, aliquoted, and frozen at −80 °C in 50% PBS, 0.05% BSA, 40% FCS (Gibco, Thermo Fisher Scientific, Zug, Switzerland), and 10% DMSO (WAK-Chemie Medical GmbH, Steinbach/Ts., Germany). The TL was analyzed in leukocytes by flow-FISH, as described by Baerlocher et al. [18]. Briefly, cells were hybridized with heat (87 °C) and 75% formamide (Millipore, Merck KGaA, Darmstadt, Germany) with fluorescent-labelled telomere-specific DNA probes complementary to the telomere repeats (Applied Biosystems, Thermo Fisher Scientific, Zug, Switzerland); the DNA was counterstained with LDS751 (Invitrogen, Thermo Fisher Scientific, Zug, Switzerland), and cell-specific epitopes were stained with CD20 (Beckman Coulter, Inc., Mississauga, ON, Canada) and CD45RA (BD Biosciences; Becton, Dickinson and Company, Allschwil, Switzerland). Telomere fluorescence was assessed by flow cytometry (FACSCalibur Flow Cytometer and FlowJo software, BD Biosciences, Becton, Dickinson and Company, Allschwil, Switzerland) and the TL values were compared to reference ranges from over 400 TL values of normal probands (aged 0–102 years) [18]. Due to the non-linear age-dependent decrease in telomere length, the age-adjusted telomere length difference (dTL) was calculated for comparison as the difference between the TL value and the age-adjusted value of the 50th percentile of the reference range. The higher the dTL, the shorter the TL adjusted for age.

### 2.4. Measurement of Telomerase Activity

An aliquot of isolated and frozen leukocytes was analyzed for TA by a protocol adapted from Mender and Shay [19]. In brief, pelleted cells were treated with a lysis buffer, and, for comparison, the protein content was adjusted to the same amount for all samples. For the telomerase reaction step, cell lysates were added to the fragment amplification reaction using a primer mix including a fluorescent (Cy5) marked primer (primer ACX 5′-GCG CGG CTT ACC CTT ACC CTT ACC CTA ACC-3′ and Cy5-TS 5′-Cy5 AAT CCG TCG AGC AGA GTT-3′, Microsynth AG, Balgach, Switzerland). To visualize the pattern of the amplified telomere fragments, the reaction volume was loaded on an acrylamide and bis-acrylamide gel (Sigma-Aldrich, Merck KGaA, Darmstadt, Germany) in a TRIS-Borat-EDTA buffer (Sigma-Aldrich, Merck, KGaA, Darmstadt, Germany) for electrophoresis (200 voltage, 1 h). A seven Cy5 dye-marked band pattern was used for analysis of the band intensities relative to the pattern of the reference [19] on each gel.

### 2.5. Ex Vivo Assays Testing the Effects of Imetelstat on Primary B-ALL Cells

Mononuclear cells were isolated from the PB of patients newly diagnosed with B-ALL (*n* = 8) by density gradient centrifugation (Pharmacia, Freiburg, Germany). Cells were incubated in Roswell Park Memorial Institute (RPMI) 1640 medium (Gibco, Thermo Fisher Scientific, Karlsruhe, Germany) containing 10% FCS (PAN-Biotech, Aidenbach, Germany), 100 U/mL penicillin, 100 μg/mL streptomycin, and 2 mM glutamine (Gibco, Thermo Fisher Scientific, Karlsruhe, Germany). Different clinically relevant concentrations (1 μM, 3 μM, and 10 μM) of imetelstat (5′-TAG GGT TAG ACA A-3′) (Geron Corporation, Foster City, CA, USA) or medium without imetelstat were added to the cultures (performed in triplicates with at least 200,000 cells at early time points and over 40,000 cells at later time points) at the time of plating. Cells were incubated for 6 to 13 days at 37 °C with 5% CO_2_ and a change in media was performed every 2–4 days. The percentage of apoptotic cells in relation to the number of viable lymphoblasts was quantified using trypan blue staining (Sigma-Aldrich, Merck KGaA, Munich, Germany) and compared to the number of apoptotic cells in relation to viable lymphoblasts in samples from the same patient incubated in the absence of imetelstat at the same time points.

### 2.6. Statistical Analyses

The demographic characteristics and results are expressed in number and percentage or median, mean, and range for categorical and continuous variables, respectively. Comparisons among the groups were performed using standard statistical tests including the Student’s *t*-test, the Pearson correlation, the rank-sum test (Mann–Whitney), and the one-way ANOVA or one-way ANOVA on ranks (Kruskal–Wallis). A *p* value < 0.05 was considered to indicate statistical significance. Data analyses were carried out with SigmaPlot for Windows version 14.0 software (Systat Software, Inc., Düsseldorf, Germany).

## 3. Results

### 3.1. Characteristics of Patients

The pediatric cohort characteristics are summarized in Table 1. In total, 18 children and adolescents with newly diagnosed ALL were included in this study. Seventeen patients were diagnosed with B-cell precursor ALL, whereas one patient had T-ALL. The median age at diagnosis was 6.5 years (range 2.2–17.8). Seven of the 18 patients were female; 11 patients were male. At the time of diagnosis, the median leukocyte count was 9.1 G/L (range 0.8–75.9), the median lymphocyte count was 1.0 G/L (range 0.6–8.1), and the median lymphoblast count was 5.9 G/L (range 0.0–35.1). The percentage of lymphoblasts in the bone marrow ranged from 70% to 100%, with a median of 97%.

**Figure 1:**
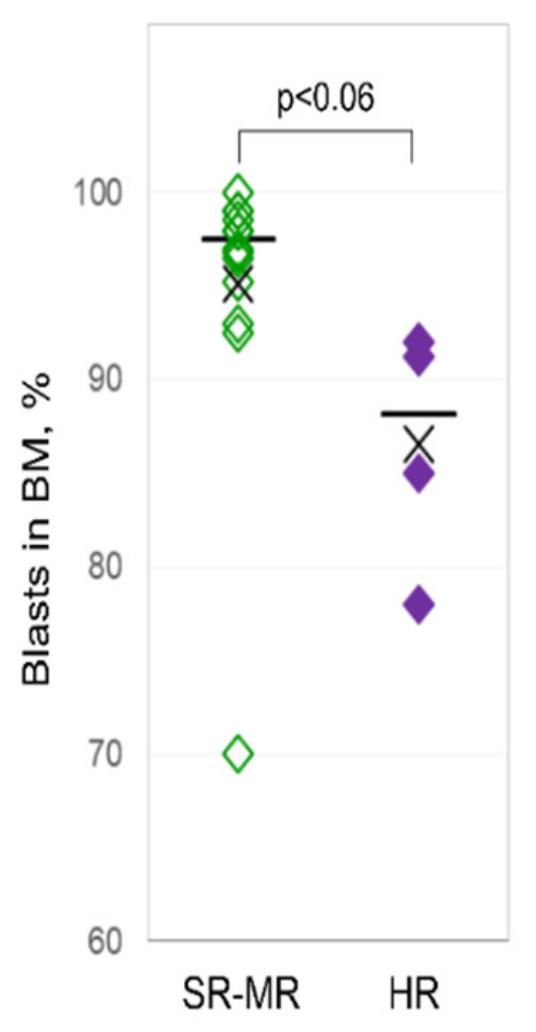
Bone marrow lymphoblasts of pediatric patients at diagnosis. Bone marrow lymphoblasts (in %) from pediatric patients with high risk (right column) compared to patients with standard and medium risk (left column) at diagnosis.

The recurrent cytogenetic aberrations comprised *ETV6-RUNX1* in six patients (33%), hyperdiploid karyotype in four patients (22%), 9p deletion in three patients (17%), and *TCF3-PXB1*-translocation in two patients (11%). Three patients (17%) carried other cytogenetic aberrations. In the final risk stratification according to the pediatric international collaborative treatment protocol of the AIEOP-BFM ALL 2009 Registry, five patients (28%) belonged to the standard-risk group, nine patients (50%) to the medium-risk group, and four patients (22%) to the high-risk group. At diagnosis, high-risk pediatric ALL patients had a lower percentage of lymphoblasts in the bone marrow (Figure 1), and a higher percentage and count of lymphoblasts in their PB compared to the non-high-risk patients (Table 1).

One child underwent allogeneic hematopoietic stem cell transplantation (HSCT) due to PCR-based risk stratification according to minimal residual disease (MRD) at indicated time points. After a median follow-up interval of 29 months (9–39), no therapy refractoriness or relapse was observed in the pediatric cohort. For additional individual patient characteristics, see Supplemental Table S1.

The adult cohort contained 14 patients, most with very high-risk characteristics at diagnosis. As the aim of the ex vivo experiments with the adult patient samples was primarily a proof of principle regarding the proapoptotic effects of imetelstat on primary human B-ALL cells, only limited clinical and laboratory data are available (see Supplemental Table S2).

## 3.2. Telomere Length

**Figure 2:**
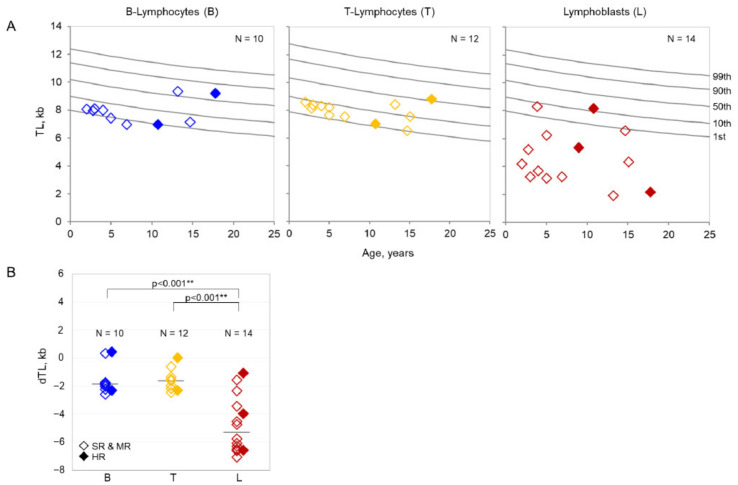
(**A**). Absolute telomere lengths of pediatric patients. Telomere lengths (TLs) in lymphoblasts (L) (**right panel**) compared to B-lymphocytes (B) (**left panel**) and T-lymphocytes (T) (**central panel**) in pediatric patients with ALL in relation to reference percentiles (grey lines) established from TLs of over 400 healthy individuals. (**B**). Age-adjusted telomere lengths of pediatric patients. Age-adjusted difference in telomere lengths (dTLs) in lymphoblasts (L) (right column) compared to B-lymphocytes (B) (left column) and T-lymphocytes (T) (central column). Pediatric patients with high risk (HR) are represented by filled diamonds, patients with standard risk (SR) and medium risk (MR) are represented by transparent diamonds. ** *p* < 0.001.

All measured TLs in lymphoblasts of patients with ALL at diagnosis were below the 50th percentile of TL reference ranges for B-lymphocytes from over 400 healthy individuals (Figure 2A). In eleven pediatric patient samples, the TL in lymphoblasts was also far below the 1st percentile of the normal reference range. The median TL in lymphoblasts was 4.3 kb (range 1.9–8.3) compared to the median TL of 8.0 kb (range 6.9–9.3) in B-lymphocytes and 8.2 kb (range 6.5–8.8) in T-lymphocytes of the same patients. The median dTL in lymphoblasts was −5.3 kb (range −7.1–−1.1) and was significantly lower (*p* < 0.001) than the median dTL in B- lymphocytes with −1.9 kb (range −2.6–0.4) and T-lymphocytes with −1.6 kb (range −2.5–0.0) in the cohort (Figure 2B). No significant linear correlations of TL with laboratory parameters, treatment response, or risk stratification were observed. Similarly, for adult ALL patient samples (*n* = 7), the median TL in lymphoblasts was 2.3 kb (range 1.9–6.2) compared to 6.3 kb (range 5.9–7.2) in B-lymphocytes and 5.7 kb (range 4.0–6.6) in T-lymphocytes (see Supplemental Table S2). The median dTL in lymphoblasts was −5.4 kb (range −5.7–−1.7) which is significantly lower (*p* < 0.001) than the median dTL in B-lymphocytes with −1.5 kb (range −1.9–0.3) and T-lymphocytes with −1.3 kb (range −3.8–0.0).

## 3.3. Telomerase Activity

**Figure 3:**
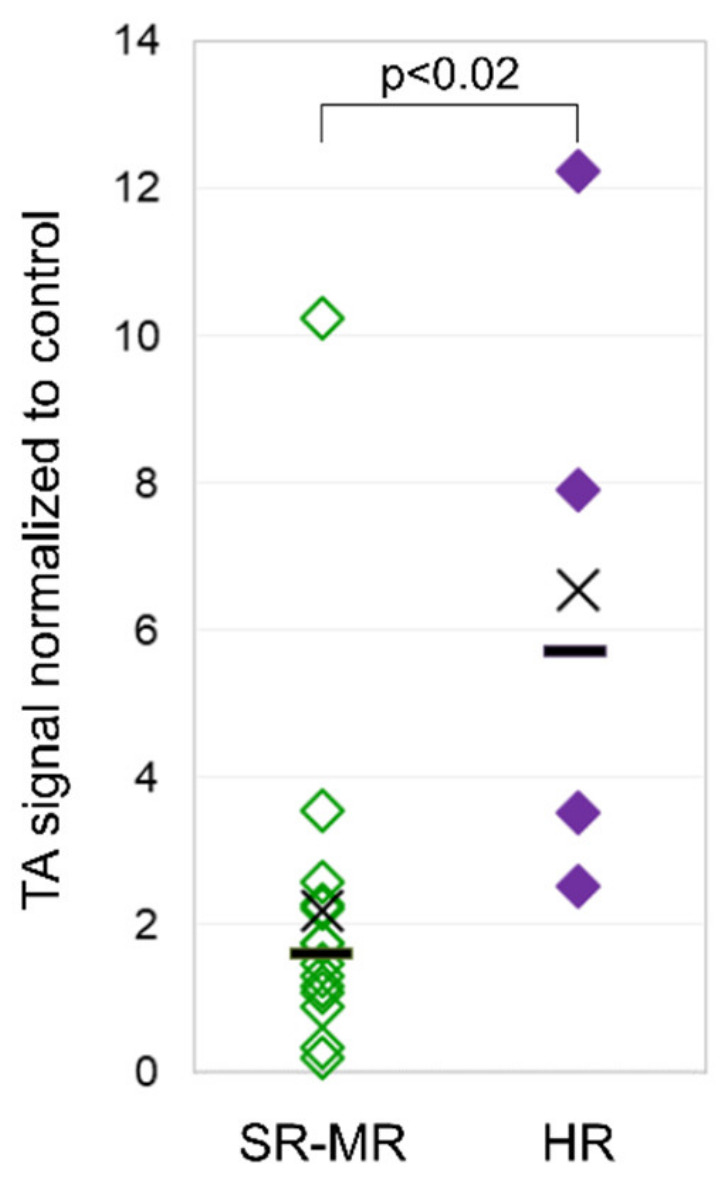
Telomerase activity in pediatric patients. Telomerase activity (TA) in leukocytes from pediatric ALL patients with high risk (right column) compared to patients with standard and medium risk (left column) at diagnosis.

The mean TA in leukocytes of pediatric ALL patients at diagnosis was 3.2 TA/C (the telomerase activity signal was normalized to control the cell line) (range 0.2–12.3), and it was 0.7 TA/C (range 0.1–1.5) for the adult ALL patients. Pediatric patients stratified as high risk showed a significantly higher mean TA of 6.6 TA/C (range 2.5–12.3) than non-high-risk patients with 2.2 TA/C (range 0.2–10.3) (Figure 3). Despite the higher TA found in high-risk patients at diagnosis, they had a rapid and steady decline in TA during the induction treatment comparable to non-high-risk patients, reflecting the lack of TA in matured, non-leukemic leukocytes in the PB (data not shown).

No linear correlation was observed between TA and other clinical or laboratory parameters. Due to the fact that all patients of the pediatric cohort responded efficiently to treatment (rapid MRD), and no relapse was observed during the study period, it was not possible to correlate TA with the uniform MRD response and outcome.

## 3.4. Ex Vivo Effects of Imetelstat on Primary B-ALL Cells

**Figure 4:**
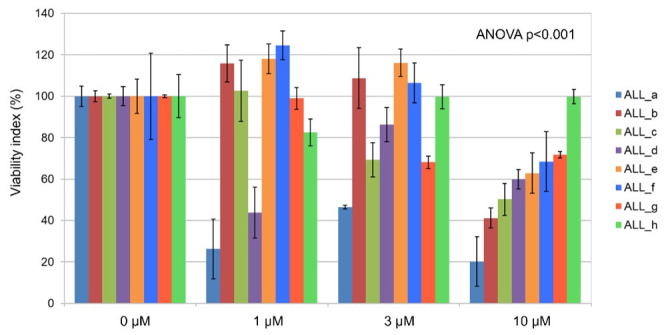
Ex vivo effects of imetelstat on primary B-ALL cells. Percentages of apoptosis in lymphoblasts (*n* = 8, ALL_a–h) treated ex vivo with different clinically relevant concentrations of imetelstat compared to lymphoblasts from the same patients cultured without imetelstat. Each panel is the result of triplicate cell cultures.

Ex vivo incubation of primary B-ALL lymphoblasts from patients with imetelstat induced significant (*p* < 0.001) dose-dependent proapoptotic effects (Figure 4). Lymphoblasts from patient samples cultured in the absence of imetelstat (=100%) compared to those cultured in the presence of 1 µM, 3 µM, or 10 µM imetelstat showed viability indices of 89.1 ± 2.6%, 87.7 ± 2.0%, and 59.3 ± 2.1%, respectively (mean ± standard error of the mean from triplicates).

## 4. Discussion

In this study, we combined the exploration of differences in telomere biology in normal versus leukemic cells within a clinical study of pediatric patients with ALL, with the investigation of ex vivo effects of the telomerase inhibitor imetelstat on primary B-ALL cells. Clinical and laboratory parameters, namely, TL and TA, were assessed for different risk groups of ALL patients. The lower lymphoblast count found in the BM and higher values in the PB of high-risk ALL patients could be due to lymphoblasts that lost adhesion molecules and migrated into the circulation and other lymphatic tissues, as has been described in solid tumors and leukemia alike [20,21].

The significantly lower TL values in ALL lymphoblasts compared to the TL values of other leukocyte subsets from the same newly diagnosed patient reflect the mitotic history of the malignant clone with an elevated number of cell divisions. Considering that human telomeres shorten by about 50–100 base pairs per cell division under physiologic conditions and up to 500 base pairs under increased oxidative stress, the median age-adjusted dTL of −5.3 kb observed in lymphoblasts of pediatric patients and −5.4 kb in adult patients corresponds to approximately 53–108, respectively 10 additional cell divisions, resulting in a high number up to 2^53–108^, respectively 2^10^ cells derived from the leukemic clone over time. Although this calculation is relatively theoretical and does not consider the short-lived nature and constant death of leukemic cells, it serves to demonstrate the immense proliferative capacity of the leukemic clone, as reflected by the short lengths of telomeres resulting from the exceptionally large number of cell divisions. The extent of clonal proliferation might even be underestimated as TA measured in ALL lymphoblasts potentially compensated, at least partially, for the loss of telomere repeats. Notably, the median size of the neoplastic clone seems to be remarkably similar for children and adults at diagnosis of ALL. Interestingly, a substantial telomere attrition was also observed in normal B- and T-lymphocytes. This loss of telomere repeats might reflect cellular proliferation induced by the anti-leukemic immune responses of lymphocyte subsets, similar to our findings in patients with CLL [22].

Earlier studies have reported telomere shortening in the blood specimens of pediatric and adult patients newly diagnosed with ALL, reflecting a subpopulation of highly proliferating cells [8,23,24,25,26,27,28]. Compared to these earlier studies, the use of the flow-FISH method, which combines the techniques of interphase FISH with cell type-specific antibody staining, allowed us to assess TL in the subtypes of cells from the same patient simultaneously without prior cell sorting.

The lower mean TA in lymphoblasts of adult ALL, in contrast to pediatric ALL, might result from a decrease in the level of TA in the stem cells and lymphocytes with age, as reported in a study of non-human primates [29]. Consequently, this lower TA in the lymphoblasts of adults compared to children and adolescents could also contribute to the even shorter lymphoblast telomeres observed in adult patients compared to pediatric patients. In general, the lack to compensate telomere attrition despite high TA could also be due to diminished or lost accessibility of telomerase to telomeres. The higher mean TA found in lymphoblasts of high-risk pediatric patients at diagnosis in this study is in line with the higher TA reported in other hemato-oncological diseases and its correlation with prognosis. Altered promotor methylation of TERT (telomerase reverse transcriptase) as well as of cyclin-dependent kinases are associated with higher TA and have been reported in patients with ALL [30,31,32]. In adult patients, TA has been established as a prognostic marker in several solid tumors and hematologic malignancies alike (e.g., non-small cell lung carcinoma and colorectal cancer [33,34], myeloproliferative neoplasms, and chronic lymphocytic leukemia [12,35]). In pediatric patients, TA has been described to correlate with the risk profile in neuroblastoma and acute myeloid leukemia [36,37]. Our data suggest that the determination of TA at diagnosis could potentially provide an additional prognostic marker in ALL, which would allow the risk profile to be assessed earlier compared to the current risk stratification mainly based on milestones over the treatment course. This approach would allow timely treatment modification and might improve the outcome in this subgroup of patients.

TA not only bears prognostic value but has become a target of new therapies through the availability of a specific telomerase inhibitor. Recent preclinical and clinical studies have shown that imetelstat, a competitive inhibitor of telomerase targeting the RNA component of TA, decreases TA and has activity in hematologic malignancies, i.e., myeloproliferative neoplasms, myelodysplastic syndromes, and acute myeloid leukemia [13,14,15,16]. In the face of efficacious pharmacologic telomerase inhibition, TA could ultimately serve as a potential additional target for the treatment of ALL, especially in patients with a high-risk profile. The dose-dependent proapoptotic effects of imetelstat on primary patient lymphoblasts observed in our ex vivo assays are suggestive of a specific sensitivity to telomerase inhibition.

A recent clinical study of imetelstat in patients with myelofibrosis showed that patients with shorter telomeres at baseline tended to have better clinical benefits, including higher spleen and symptom responses and longer overall survival, compared to patients with longer telomeres [38]. The results from the present study showed significantly lower TL values in ALL lymphoblasts, suggesting that ALL may represent a suitable type of cancer for imetelstat treatment.

It is intriguing to consider that imetelstat might complement bortezomib, which has recently been implemented in the treatment of high-risk ALL patients, and in addition to proteasome inhibition, also targets telomerase integrity [39,40]. The latter mechanism may even potentiate the effect of direct telomerase inhibition by imetelstat.

This pilot study comprised a limited number of patients with high-risk ALL and, therefore, further data from pediatric and adult ALL patients are needed to validate these results. Our prospective pilot study, however, illustrates that the assessment of TL and TA can be easily integrated into larger trials and, eventually, into clinical routine diagnostics. These techniques are particularly attractive for use in pediatric patients as they also maintain a high sensitivity with small sample sizes only containing very few target cells.

## Figures and Tables

**Table 1:** Demographic, hematologic, cytogenetic, and response data of 18 pediatric patients with acute lymphoblastic leukemia (ALL).

**Characteristics at Diagnosis of ALL**	**Overall (n = 18)**	**Non-High Risk** **(n = 14)**	**High Risk (n = 4)**
**Age, median years (range)**	6.5 (2.2–17.8)	5.6 (2.2–15.1)	9.9 (5.8–17.8)
**Sex (f/m)**	7/11	6/8	1/3
**Hematology**			
**Leukocyte count in PB (G/L) median (range)**	9.1 (0.8–75.9)	7.7 (0.8–75.9)	21.9 (5.7–32.3)
**Lymphocyte count in PB (G/L) median (range)**	2.0 (0.6–8.1)	2.0 (0.6–8.1)	2.0 (0.7–2.3)
**Lymphoblast count in PB (G/L) median (range)**	5.9 (0.0–35.1)	4.1 (0.0–35.1)	17.1 (3.2–27.8)
**Lymphoblast proportion in PB (%) median (range)**	68 (0–90)	64 (0–90)	79 (56–87)
**Lymphoblast proportion in BM (%) median (range)**	97 (70–100)	98 (70–100)	88 (78–92)
**Cytogenetics**			
**t(12;21)/ETV6-RUNX1**	6	6	0
**Hyperdiploidy**	4	4	0
**Deletion 9p + other aberration**	3	3	0
**t(1;19)/TCF3-PXB1**	2	2	0
**Other**	3	0	3
**Treatment response**			
**Blast count in PB day 8 (G/L) median (range)**	0.028 (0–0.600)	0.028 (0–0.500)	0.181 (0.003–0.600)
**FACS-MRD in BM day 15 (%) median (range)**	0.30 (0–14.18)	0.30 (0–5.40)	5.63 (0.03–14.18)
**PCR-MRD BM day 33 (log) median (range)**	1.0 × 10^−6^ (neg. −1.0 × 10^−3^)	1.0 × 10^−6^ (neg. −2.0 × 10^−4^)	7.0 × 10^−4^ (neg. −1.0 × 10^−3^)

## Data Availability

All data generated during this study were analyzed and summarized in this published article and the Supplementary Materials.

## Supplementary Materials

The following are available online at https://www.mdpi.com/article/10.3390/ijms22136653/s1.
