# Supplementary material for: Targeting Telomere Biology in Acute Lymphoblastic Leukemia
Source: Int J Mol Sci. 2021 Jun 22;22(13):6653. doi: 10.3390/ijms22136653 (PMC8268026; doi:10.3390/ijms22136653)
Supplement: Supplementary file 1 [file ijms-22-06653-s001.zip › ijms-1221425-supplementary.pdf]

Supplementary Materials:

Supplemental Table S1A-C

Demographic, hematologic and cytogenetic findings (A), response to treatment, risk stratification (B), TL and TA (C) for the individual pediatric patients. n.a. = not available

A

ID	Sex	Age at diagnosis (y)	Hemoglobin (g/l)	Leukocytes (G/L)	Platelets (G/L)	Neutrophils (G/L)	Lymphocytes (G/L)	Blasts in PB (%)	Blasts in PB (G/L)	Blasts in BM (%)	Cytogenetics/FISH
AL01	f	9.0	83	19.3	20	0.2	2.3	87	17	78	47 XX +21
AL02	m	10.8	79	5.7	137	0.3	2.1	56	3	85	near haploidy
AL03	f	2.8	104	7.0	117	0.2	1.2	78	5	98	high hyperdiploidy
AL04	f	13.2	104	38.6	184	3.7	3.1	81	31	97	9p-, 13q-
AL05	f	6.9	89	1.4	27	0.2	0.6	15	0	70	translocation t(12;21)
AL06	m	17.8	154	24.5	86	4.8	2.0	71	17	92	Ph+, translocation t(9;22)
AL07	m	14.7	113	9.8	17	0.6	8.1	65	6	99	translocation t(1;19)
AL08	m	15.1	63	2.8	19	0.0	0.8	63	2	98	9p-, t(2;16)
AL09	m	3.9	93	10.4	35	0.3	1.8	78	8	93	translocation t(12;21)
AL10	f	5.2	92	0.8	171	0.1	0.7	0	0	100	9p-, high hyperdiploidy
AL11	m	2.2	84	8.3	48	0.2	4.9	0	0	97	translocation t(12;21)
AL12	m	7.6	67	23.1	57	0.7	1.3	90	21	97	translocation t(1;19)
AL13	f	4.8	67	5.4	20	0.5	2.2	0	0	93	high hyperdiploidy
AL14	m	5.8	72	32.3	81	0.8	0.7	86	28	91	complex aberrant
AL15	m	4.2	82	75.9	79	5.3	3.4	72	35	98	translocation t(12;21)
AL16	f	13.9	65	1.4	122	0.4	1.0	1	0	99	high hyperdiploidy
AL17	m	3.8	75	5.3	104	0.1	2.5	51	3	99	translocation t(12;21)
AL18	m	6.0	97	19.9	30	0.4	3.0	81	16	95	translocation t(12;21)

B

ID	Blast Count in PB day 8 (G/L)	FACS-MRD day 15 (%)	PCR-MRD day 33 (log)	Risk Stratification
AL01	0.060	11.20	positive 4.0 E-4	HR
AL02	0.301	0.03	negative	HR
AL03	0.002	0.30	positive 1.0 E-6	SR
AL04	0.012	5.40	positive 1.0 E-6	MR
AL05	0.000	0.00	negative	SR
AL06	0.003	0.07	positive 1.0 E-6	HR Ph+
AL07	0.067	1.79	negative	MR
AL08	n.a.	0.08	positive 1.0 E-6	MR
AL09	0.121	0.16	negative	SR
AL10	0.000	n. a.	positive 2.0 E-4	MR
AL11	0.000	0.38	positive 1.0 E-6	MR
AL12	0.026	0.11	positive 1.0 E-6	MR
AL13	0.440	1.19	positive 1.0 E-6	MR
AL14	0.600	14.18	positive 1.0 E-3	HR
AL15	0.500	0.17	negative	SR
AL16	0.028	0.98	negative	MR
AL17	0.107	0.05	positive 1.0 E-6	MR
AL18	0.028	1.18	negative	SR

C

ID	T-Lymphocytes TL (kb)	B-Lymphocytes TL (kb)	Lymphoblast TL (kb)	TA/C at T0
AL01	n.a.	n.a.	5.3	12.3
AL02	7.0	6.9	8.2	2.5
AL03	8.1	8	5.2	2.2
AL04	8.4	9.3	1.9	10.3
AL05	7.5	7.0	3.3	0.9
AL06	8.8	9.2	2.2	7.9
AL07	6.5	7.1	6.6	1.7
AL08	7.5	n.a.	4.3	1.5
AL09	n.a.	n.a.	8.3	1.1
AL10	n.a.	n.a.	n.a.	1.2
AL11	n.a.	n.a.	n.a.	2.3
AL12	8.6	8.1	4.2	1.3
AL13	8.2	n.a.	6.2	0.3
AL14	n.a.	n.a.	n.a.	3.5
AL15	8.3	8.0	3.7	3.6
AL16	n.a.	n.a.	n.a.	0.2
AL17	8.4	8.1	3.3	2.6
AL18	7.6	7.4	3.2	1.7

Supplemental Table S2

Demographic, hematologic parameters, cytogenetics, and risk stratification (A), TL, TA and viability index (B) for the individual adult patients. n.a. = not available

A

ID	Sex	Age at diagnosis (y)	Hemoglobin (g/l)	Leukocytes (G/L)	Platelets (G/L)	Neutrophils (G/L)	Lymphocytes (G/L)	Blasts in PB (%)	Blasts in PB (G/L)	Immunphenotype	Cytogenetics/ FISH	Risk stratification *
ALL_a	f	60	10.5	54.0	174.0	1.4	12.4	86.0	39.0	common B-ALL	t(9;22)(q34;q11)	HR
ALL_b	m	26	15.0	123.0	124.0	n.a.	n.a.	n.a.	n.a.	common B-ALL	del(5)(q13)	VHR
ALL_c	f	50	n.a.	n.a.	n.a.	n.a.	n.a.	n.a.	n.a.	mature B-ALL	+8	VHR
ALL_d	f	37	8.2	130.0	29.0	5.2	13.0	86.0	112.0	common B-ALL	46, XX	VHR
ALL_e	f	18	10.2	50.2	29.0	6.0	8.8	38.0	19.0	relapsed common B-ALL	n.a.	n.a.
ALL_f	f	73	8.5	220.0	22.0	n.a.	n.a.	n.a.	n.a.	n.a.	n.a.	VHR
ALL_g	f	80	n.a.	122.0	n.a.	n.a.	n.a.	90.0	n.a.	relapsed pro-B-ALL	46, XX	VHR
ALL_h	m	82	n.a.	n.a.	n.a.	n.a.	n.a.	n.a.	n.a.	n.a.	n.a.	n.a.
ALL_i	f	60	8.0	17.0	31.0	0.2	7.2	n.a.	n.a.	pro-B-ALL	46, XX	VHR
ALL_j	m	32	8.9	126.0	21.0	11.3	15.1	78.0	98.0	common B-ALL	46, XY	VHR
ALL_k	f	37	9.7	10.6	207.0	1.7	1.9	65.0	7.0	relapsed common B-ALL	46, XX	VHR
ALL_l	f	56	8.5	24.1	92.0	16.9	5.8	21.0	5.0	common B-ALL	t(8;14) (q24;q32)	VHR
ALL_m	f	63	9.9	106.0	68.0	6.4	11.7	74.0	78.0	mature B-ALL	46, XX	VHR
ALL_n	m	49	8.7	150.1	20.0	n.a.	n.a.	n.a.	n.a.	common B-ALL	46, XY	VHR

* based on available parameters at diagnosis

B

ID	T-Lymphocytes TL (kb)	B-Lymphocytes TL (kb)	Lymphoblast TL (kb)	TA/C at T0	Viability index 1μM (%)	Viability index 3μM (%)	Viability index 10μM (%)
ALL_a	4.9	5.9	2.0	0.4	26	47	20
ALL_b	n.a.	n.a.	n.a.	1.2	116	109	41
ALL_c	5.7	7.0	2.3	1.5	103	69	50
ALL_d	n.a.	n.a.	n.a.	n.a.	44	86	60
ALL_e	n.a.	n.a.	n.a.	n.a.	118	116	63
ALL_f	5.3	n.a.	1.9	0.4	125	107	69
ALL_g	n.a.	n.a.	n.a.	n.a.	99	68	72
ALL_h	n.a.	n.a.	n.a.	1.2	83	100	100
ALL_i	6.6	7.2	3.1	0.1	n.a.	n.a.	n.a.
ALL_j	4.0	6.2	3.5	0.3	n.a.	n.a.	n.a.
ALL_k	6.6	6.4	2.2	0.3	n.a.	n.a.	n.a.
ALL_l	n.a.	n.a.	n.a.	0.5	n.a.	n.a.	n.a.
ALL_m	n.a.	n.a.	n.a.	1.4	n.a.	n.a.	n.a.
ALL_n	5.7	6.1	6.2	n.a.	n.a.	n.a.	n.a.
